# Investment Subsidies Effectiveness: Evidence from a Regional Program

**DOI:** 10.1007/s40797-022-00196-1

**Published:** 2022-06-16

**Authors:** Simone Chinetti

**Affiliations:** Department of Economics and Statistics, University of Salerno, Via Giovanni Paolo II, 132, 84084 Fisciano, SA Italy

**Keywords:** Innovative investments, Public subisidies, SMEs, Regional policy, H25, L52, O31, O38, R58

## Abstract

I provide novel empirical evidence on the effectiveness of public funding in lagging behind areas by investigating the effect of a subsidy program implemented in the Campania region (South Italy) and targeting SMEs. By relying on a Difference-in-Differences approach, my estimates demonstrate that the regional program produces a sizable increase in private firms’ innovative investment spending. However, I show also large heterogeneity in the firms’ response. In particular, I find that the positive effect on investment, compatible with the input-additionality hypothesis, comes from medium-large firms and low-tech medium-large enterprises. Finally, I show that the program has considerable indirect effects on medium-large low tech service firms’ labour demand but not on overall firms’ productivity.

## Introduction

Most developed countries have economic policies to revive and balance growth, especially targeting lagging behind areas. Italy is not an exception in this sense, given the dramatic and structural divide between the northern and southern regions of the country.

First, the Great recession and then the COVID-19 pandemic have renewed the interest in the economic effects of public policies. Motivated by tight budget constraints, the importance of understanding whether these place-based policies accomplish their goals has grown significantly. Infrastructure investment, incentives to boost labour market participation and human capital, and subsidies to enterprises to innovate, move or remain in underdeveloped areas are usual features of these programs.

In this paper, I provide novel empirical evidence on the effectiveness of place-based policies in lagging areas by studying whether public funding foster private firms investment in innovation. The objective of the analysis, thus, is to evaluate if regional funds can generate additional effects on innovative capital investments above those foreseen by the market.

Public funding is usually aimed at influencing the allocation of investments and employment to improve competitiveness, growth and labour market dynamism in disadvantaged regions. Indeed, policymakers use financial incentives to change firms preferences and to push them to invest in projects that, without contributions, would be usually be abandoned. However, this complementary mechanism is far from occurring. The problem of additionality of public aids traces back to the presence of asymmetric information between the central governments, or the local administration in charge of managing the funds, and firms: if the policymaker knew the minimum incentive necessary to activate any (new) investment, the complementary mechanisms would be maximized, and the deadweight loss would be negligible.

To draw causal evidence, I leverage the implementation of a regional subsidy program to foster innovative investments for small and medium firms (SMEs) in the Campania region, one of the Italian lagging behind areas, during 2014–2015. Specifically, firms were invited to submit proposals for new innovative projects. Only those scoring above a certain threshold received a subsidy, covering up to $50\%$ of expenditures in innovative intangible and tangible assets according to the selection criteria set by the region. One of the interesting aspects of this regional policy, whose selection is based on competitive rankings, is that it is possible to build a control group based on the firms that applied to the program but are not among the “winners”. As highlighted by the literature, the use of this control group should strengthen the empirical analysis (Bernini and Pellegrini 2011). Indeed, the rejected pool of applicants may be close to a control group since it comprises firms that are sufficiently similar to the treatment group in terms of characteristics and includes eligible companies willing to receive the funds (Brown et al. 1995).

Furthermore, this regional program qualifies itself as an appealing case study for at least three reasons. First, the program rules require that firms willing to be financed in developing innovative activities must operate (and be located) within the region boundaries. Hence, the policy’s local dimension allows me to remove much of the unobserved heterogeneity among enterprises that, instead, characterizes nationwide programs by comparing a sample of more homogeneous firms (subsidy-recipients and non-subsidy-recipients) based and operating in the same region and thus exposed (reasonably) to the same set of business rules and local shocks. Then, an additional program requirement obliged participating firms to request funding for brand new investment projects and develop them only with regional support (subsidy). That is, the program rules forbid firms from combining several public incentives. In this way, I am more confident of estimating a clean causal effect (if any) that comes only from the effect of regional subsidies on the level of innovative investment. Finally, the regional government pledged sizeable funds to foster investments in private firms. Indeed, about 22 million euros of public resources have been distributed to firms to induce them to increase innovative expenditures.

To recover the causal effect of interest, I exploit detailed data on the regional program matched with balance sheet data from the AIDA database (managed by Bureau Van Dijk) for treatment and control groups. I employ a Difference-in-Differences approach by comparing the average innovative capital investment of subsidy recipients and non-recipient firms, identified by taking advantage of the assignment scheme of the regional funds before and after the program implementation.

According to my estimates, the regional subsidy program has a positive, sizable and statistically significant effect on eligible firms’ capital investment in innovation. The coefficient measuring the causal effect of interest is about 51 thousand euros, translating into a notable relative increase in innovative capital of 1.5 times higher than the 2013 average investment level. Furthermore, by relying on a back-of-the-envelope calculation, I estimate an implied elasticity of 3 - a value somewhat higher but in the range of those documented in the literature (Hall and Van Reenen 2000). In addition, the sizable increase in innovative capital is compatible with the input-additionality mechanism since I cannot accept the hypothesis that treated firms increased spending by about the size of the regional funds they received.

Also, I show considerable heterogeneity in the firms’ responses. First, I show that different levels of the awarded subsidy (proxied by the quartiles of its distribution) produce an inverted U-shaped investment response. Then, I conduct an in-depth analysis to understand to what extent firm size, technology level, and economic sectors may play a role in shaping the effect of the subsidy on innovative capital investment. Differently from the main findings in the literature, the heterogeneity analysis suggests that the additionality effect of the subsidy materializes only for medium-large firms and medium-large low-tech companies, with a relative increase in innovative capital of $+100\%$ and $+120\%$, respectively.

Moreover, I perform a series of robustness checks showing that these results are adequately consistent across small bandwidth sizes around the threshold eligibility. Finally, I document that the program has a considerable indirect effect on low-tech medium-large service firms’ labour demand but not overall improvements in productivity.

This paper has ties to two main strands of the literature. The first, and most related one, regards the empirical micro-evidence on the effectiveness of public programs in underdeveloped areas. For decades, economists have been debating the extent to which investment incentives have an economic payoff (see, for instance, King 1977 and Hall and Jorgenson 1969). Further, the regional science literature considers a significant issue of whether local iniquities can be ameliorated through public incentives (Harris and Trainor 2005; Gabe and Kraybill 2002; Glaeser 2001; Faini and Schiantarelli 1987). Despite a large body of research, few have focused on the effectiveness of investment incentives to firms located in lagging areas. Besides some recent studies’ increased optimism, findings remain mixed. Some studies show that capital incentives can prompt additional investment in subsidized firms (Criscuolo et al. 2019; Bondonio and Greenbaum 2014; Schalk 2000; Daly et al. 1993; Harris and Trainor 2005; Faini and Schiantarelli 1987), while others suggest intertemporal substitution effects (Bronzini and de Blasio 2006). Also, the impact of investment incentives on employment is doubtful, while that on productivity appears negligible (or even negative).

About the Italian context, most of the available evidence has focused on the impacts of Law 488/1992. This regional policy has been the primary industrial policy targeted to manufacturing and extractive firms willing to invest in lagging areas. Overall, these studies show that the Law 488/1992 subsidies have positive effects on output, employment and fixed assets (at least in the short run) but a less significant increase in total factor productivity (see among the many Cerqua and Pellegrini 2014 and Bernini and Pellegrini 2011). In that respect, my study complements the available evidence and enriches it since my setting consists of a sample of potentially eligible firms operating in all economic sectors rather than in manufacturing and extractive industries only, despite being restricted only to one area of the country.

Also, qualitative reviews of the literature, both at the international (Becker 2015; Zúñiga-Vicente et al. 2014) and national levels (Bocci et al. 2021; Cerqua and Pellegrini 2020), confirm the extreme heterogeneity in results that further require investigation. The available evidence suggests a tendency to find positive effects on firms’ employment, investment and survival, mostly limited to small firms and enterprises in low-tech sectors, while questionable effects on productivity.

Finally, the second strand of the literature to which this paper is related regards the management and allocation of public resources. Indeed, this study permits to shed light on the effects of place-based policies managed by local governments, that, however, have always had scant attention from the impact evaluation literature, despite the prominent role of local governments in shaping the local economic conditions and the relatively great bulk of public resources that the private sector absorbs from the public sector (Kline 2010).

The remainder of the paper is structured as follows. In Sect. 2, I illustrate the features and characteristics of the investment subsidy program, by also describing qualitatively the technological nature of the subsidized projects. Section 3 describes the data and the empirical strategy used to recover the causal effect of interest. In Sect. 4, the main results are discussed. In Sect. 5, the estimates relative to the heterogeneity analysis are presented. Section 6 presents some robustness checks. In Sect. 7, I explore whether the program had indirect effects on other firms’ outcomes. Finally, Sect. 8 concludes.

## The Regional Program

In 2009, the Government of Campania published the “Regional SMEs Support Program de Minimis for Organizational, Process and Products Innovation” (ex Reg. (CE) No. 1998/2006), a regional program, with an endowment of more than 20 millions of Euro, intended to sustain through public monetary support, in the form of direct subsidies, private brand new innovative investment developed by requesting small and medium firms with particular regards to those connected with information technology.

The priority was to favour the implementation of innovative investment programs, through the use of new technologies (ICT in particular), by improving the competitiveness of the local business fabric and increasing the productivity of the same firms, also from a management and product innovation point of view.

Specifically, firms were invited to submit proposals for new projects, and the regional government subsidized the innovative investment expenditures of eligible firms through a direct grant (subsidy). The grant may cover up to $50\%$ of the costs for intangible and tangible innovative assets and $10\%$ for expenses connected to the project’s development. In any case, the maximum grant per project cannot exceed the sum of 200 thousand euros to avoid any conflict with the European State Aid Legislation. Eligible firms, including temporary associations or consortia, were those firms that had the main operative office located in the region and, most importantly, intended to implement the project within the regional boundaries.

The subsidy covered the following investment outlays: (a) research and development expenditures, (b) start-up and expansion costs, (c) patents, (d) licenses et similar rights, (e) plant and machinery and (f) industrial and commercial equipment.

**Fig. 1:**
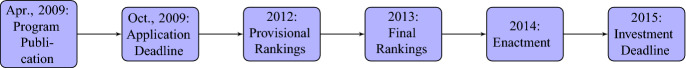
Regional program timeline

Even though the program’s call expired in 2009, its implementation took place only in 2013, and eligible firms were to put in place investments during a two years window (2014–2015), while subsidies were materially transferred to eligible firms throughout 2014 (see Fig. 1).

A critical characteristic of the program was that firms could not receive other types of public subsidies for the same project. This requirement helps the evaluating process, given that the impact of the regional program cannot be confused with that of other public subsidies. In addition, all the projects must be brand new since no eligibility was granted for projects that involved the completion of old investments started prior to submitting the proposal to the region.

The grants were assigned after a process of projects’ assessment carried out by a Technical Commission appointed by the Regional Government. The commission examined the projects and assigned a score for each of the following criteria: (a) Project Quality and Innovation (*max* 60 pts), (b) Competitiveness and Impact on Product/Service (*max* 30 pts) and (c) Youth and Female SMEs (*max* 10 pts).^1^ Proposed projects obtaining a total score equal to or greater than 60 points (max score 100 pts) received the grant. For the evaluation process, the Technical Commission must comply with the general principles for the research evaluation specified by the Ministry of Education, University and Research of the Italian Government, and the European Commission’s general principles.

**Table 1:** Investment programs evaluation and criteria

Criteria	Score
Project quality and innovation	*max* 60 pts
Competitiveness and impact on product/service	*max* 30 pts
Youth and female SMEs	*max* 10 pts
Total	100/100
Min Score ($\rightarrow$ subsidy)	60/100
Final ranking results	
Treated firms ($score\ge 60$)	299
Control firms ($score<60$)	424

Assessment of innovative projects are carried out by a Technical Commission appointed by the Regional Government according to the general principles for the research evaluation specified by the Ministry of Education, University and Research of the Italian Government and the general principles of the European Commission

**Table 2:** Subsidy summary statistics

	Mean	SD	Min	Max
$SUBSIDY_{i2014}$ ($\%$)	43	6.78	20.8	50
$SUBSIDY_{i2014}$	79,979.8	50,282.87	6,871.5	200,000
$SUBSIDY_{i2014}/TA_{i2013}$ ($\%$)	8.47	15.87	.147	125.6
$INV_{i2013}/TA{i2013}$ ($\%$)	1.23	4.78	0	50.39

*SUBSIDY* *i*2014 is expressed in units of Euro

At the end of 2009, when the program application deadline expired, 2174 firms requested access to the public grant. However, when the program results were released in 2013, just 396 of the 2174 requesting firms were eligible, leaving 424 non-recipients and about 1354 companies excluded from the program participation.^2^ Overall, the region has granted to eligible firms about €22 million, meaning that it has committed to finance, on average, about $43\%$ of total spending in innovative investments. The average treated firm received about €79,900 (see Fig. 6 for the average subsidy awarded by score and Table 2). In addition, the subsidy value amounted to 8 percent of total assets in 2013 (see Table 2), while during the same year, the share of innovative investment over total assets was about, on average, $1.2\%$.

As I have already discussed in the previous lines and shown in Fig. 1, the program was published in the Regional Journal (Bollettino Ufficiale della Regione Campania, BURC^3^) in April 2009, but its actual enactment only occurred during 2014–2015, after the publication of the final ranking list of eligible firms by the regional government in late 2013.

Given the 5 years between program publication and implementation, one may argue that projects that could have been considered as *innovative* in 2009, in 2014 may result out-dated since technological advancements and improvements have occurred (given, also, the dramatic shortening of the technology life-cycle). So, from my point of view, apart from the program evaluation exercise to assess the additional effect of the subsidy on the level of innovative investment, it appears very useful to discuss the technical nature of the projects conducted by *recipient*-firms. In order to accomplish this task, I compare (at random) some of the firms’ projects that obtained the lowest possible score to be declared eligible in obtaining the public support (*score* :  60–61) with that of firms that, instead, scored the highest ($score>71$).

According to the official documents and projects released by the Campania region (available only for *recipient*-firms), low-scoring firms presented innovative projects involving the adoption of *Enterprise Resource Planning* (ERP) systems along with the creation of websites to manage service provision with clients and *e-commerce* platforms.^4^ Nowadays, ERP systems are widely diffused among enterprises, still very useful to manage global business processes, and their adoption still contributes to improvements and reinforcement in a firm’s competitive advantage. Furthermore, the creation of websites and *e-commerce* platforms are crucial instruments available to businesses to increase their chance of broadening the base of customers, becoming more visible on the market and encouraging them to shape and adopt business strategies oriented towards the penetration of international markets given that Internet allowed for the breaking down of physical boundaries.

On the other hand, high-scoring firms proposed projects that, along with the adoption (or renovations) of ERP systems as well as the creation of *e-commerce* platforms, also included innovative activities aimed at improving substantially, especially from a technological point of view, the product/service they were offering on the market. For example, one of the two high-scoring firms I chose to write this paragraph stated that if the subsidy had been awarded, a consistent portion of the money would have gone toward the installation of digital sensors on plants, allowing the company to track production in real-time and communicate with other plants and business sectors to improve the overall process. Alternatively, the latter enterprise, in order to improve its position on the international marketplace, was planning to develop IT programs able to standardize the software development process in order to reduce its costs and implementation time and be competitive with foreign companies while still offering superior quality and highly specialized service to its customers.

To sum up, the lag between the program publication and its implementation did not seem to have played a role in reducing the program’s technological improvements. On the contrary, apart from the heterogeneity on the level of technological advancements proposed by firms that are also able to explain the differences in reported scores (at least in part), all of them submitted projects involving the adoption of technologies that were not meant to become obsolete within a few years.

## Data and Empirical Strategy

*Sample and Descriptive Statistics* The empirical analysis builds on an original and novel dataset combining two sources of information. The regional investment subsidy program data are retrieved from the Campania Region website. This dataset reports information on recipients and non-recipients firms, such as company name, tax code number, score received, planned investment (only for subsidy awarded firms), grant assigned, subsidies revoked and renunciations. Then, I combine these information with balance sheet data covering several firms’ dimensions over 2008–2016. The source of firm-level data is AIDA, a database produced by Bureau van Dijk that collects balance sheet information on all Italian firms required to file it; the requirement applies to corporations but not to partnerships.

By matching the firms’ tax code number, I can retrieve 232 out of 299 (net of renunciations) enterprises that obtained a score $\ge 60$ and 313 out of 424 control firms from AIDA. Unfortunately, the rescued data cannot be considered a random sample since AIDA does not collect balance sheet information on partnerships, and that is why the overall number of firm data is lower than the total number of treated firms resulting from the official regional records. In addition, as summarized by Table 14, the sample further shrinks in size because of bankruptcies, liquidations and dissolutions (including voluntary closures) or because the firms (for unknown reasons) are no longer reported in the database. However, this sample reduction can be considered unrelated to program participation/non-participation. First, the awarded subsidy cannot finance other business activities other than the planned investment, and it is unlikely that a firm survives in the post-implementation period because of the regional funds (whose average amount is relatively low). Second, both treated and control firms during the post-intervention period experienced these events. Finally, in many cases, such extraordinary procedures began earlier than the regional program, and during this period, balance sheet quality is severely affected (i.e. many financial statement items start to be missing or reported with unusual values).

Hence, to avoid attrition and data quality concerns, I conduct the empirical analysis considering only those firms for which data are available up to 2016. Furthermore, I restrict the analysis’ time horizon from 2010 to 2015, 3 years as pre-treatment period and 2 years as post-implementation period (since firms were allowed to put in place investment from January 2014 to December 2015). Then, the final dataset results in an almost perfectly balanced sample composed of 182 subsidy recipient firms and 186 enterprises who failed to obtain the regional funds (${\mathrm{score}}<60$).

The paper aims to estimate the causal effect of the regional subsidy on private firms’ innovative investment. Regrettably, the AIDA database does not provide information on firms’ investment flows. For this reason, I rely on a proxy for the true firms’ investment closely following Cerqua and Pellegrini (2014). In particular, my investment measure is given by the yearly changes of tangible and intangible capital.^5^

Hence, the outcome variable reflects the overall yearly variation of the intangible ($R \& D$ expenditures, Patents, Licenses et similar rights) and tangible (Plant and Machinery and Industrial and Commercial Equipment) innovative investments for which the region assigned the subsidy, given that treated firms may spend in tangible innovative assets only, in intangible innovative assets only or both.

**Fig. 2:**
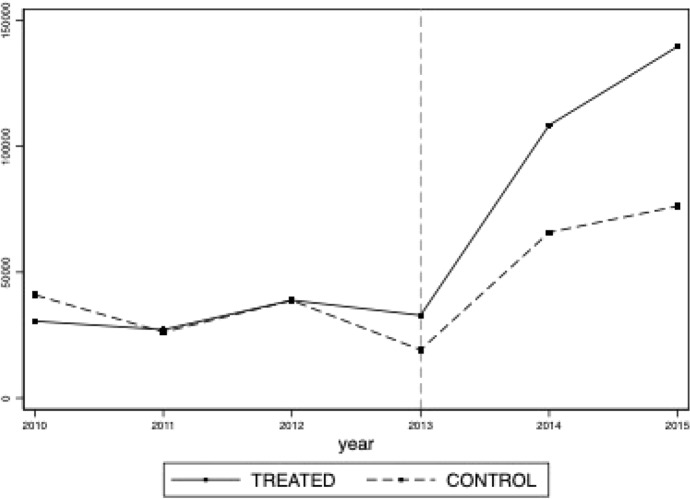
Average firms’ investment

Figure 2 compares the evolution of the outcome variable during 2010–2015 for treated and control groups. During the three pre-treatment years, innovative spending has followed a very similar dynamic for both groups of firms. Instead, subsidy and non-subsidy recipient firms increased spending during the program implementation period, but that of treated firms increased more and peaked at its maximum in 2015, hopefully, because of the subsidy.

*Empirical Strategy* The objective of the empirical analysis is to assess the causal effect of public subsidies on firms’ innovative expenditures. As argued in Sect. 2, the subsidy program implemented in the Campania region has a crucial feature that aids the evaluation: firms cannot get other public funds for the same investment project. This condition minimizes other potential sources of bias since the regional program’s impact cannot be confounded with that of other government incentives. However, I cannot exclude that firms may request other incentives to finance other investment projects. To correctly identify the counterfactual impact estimates throughout the rest of the paper, I assume that the probability of firms gaining access to other unobserved regional or national program incentives is the same for both groups of firms.

Public funds are provided to requesting firms only for those investment projects that obtain a minimum score of at least 60 points after the Technical Commission’s evaluation process. Therefore, the treatment assignment is based on a (sharp) deterministic rule that, as explained in Sect. 2, firms cannot manipulate nor influence. In particular, the assigned score determining treatment eligibility is based on a competitive projects’ ranking that rewards for a $90\%$ of the overall score proposals’ quality and competitiveness and for the remaining $10\%$ specific firms’ characteristics measured in 2008 (such as shareholders’ (average) age and prevalent sex and projects’ economic sustainability intended as how much of the planned costs are covered by firm sales volume).

Given this framework, the straightforward empirical strategy to estimate the causal effect of interest is given by a sharp Regression Discontinuity Design. However, a few limitations prevent me from exploiting this methodology. First, the RDD identification assumption requires no other jumps in a neighbourhood the cut-off other than the main outcome variable. Table 15 shows that the balancing between firms who barely obtained the subsidy with those who barely failed is not satisfied, even in tighter bandwidth size around the eligibility threshold. In addition to that, the RDD methodology works well with cross-sectional data. Instead, my setting has a panel structure since eligible firms have a two-year window to put planned investment in place, creating uncertainty on how to specify the model correctly. Finally, other complications originate from the delayed program implementation and the advent of the financial crisis. For all these reasons, I rely on a Difference-in-Differences approach and following the assignment scheme of the subsidy I define treated firms all those with score$\ge 60$, whereas controls those with ${\mathrm{score}}<60$ (as suggested by Bernini and Pellegrini (2011) and Brown et al. (1995)).

**Table 3:** Summary statistics

	Control	Obs.	Treated	Obs.	$\Delta$
*Year = 2008*					
INVESTMENT	134,497.67	153	78,980.34	152	55,517.33
LEVERAGE	8.17	169	9.34	168	− 1.17
COST OF DEBT	8.47	75	8.97	80	− 0.50
ROA	4.95	169	7.63	168	− 2.68$^{*}$
SALES	3,581,637.49	169	5,420,068.76	168	− 1,838,431.27
EBITDA	269,125.64	169	483,065.65	168	− 213,940.02$^{*}$
CASH FLOWS	153,537.07	169	266,020.53	168	− 112,483.46
LIQUIDITY	209,174.35	169	297,536.65	168	− 88,362.31
NO. WORKERS	32.57	140	21.04	139	11.53

*Years = 2010–2013*					
INVESTMENT	31,258.22	699	32,357.21	715	− 1099.00
LEVERAGE	7.93	716	7.12	719	0.80
COST OF DEBT	7.86	391	7.66	434	0.20
ROA	4.00	719	5.43	719	− 1.44$$^{**}$$
SALES	3,775,624.97	719	5,926,232.16	719	− 2,150,607.19$$^{***}$$
EBITDA	263,791.98	716	547,793.44	719	− 284,001.46$$^{***}$$
CASH FLOWS	152,060.22	716	349,701.69	719	− 197,641.47$$^{***}$$
LIQUIDITY	244,545.78	716	322,573.59	719	− 78,027.81
NO. WORKERS	26.71	675	23.07	679	3.64

Statistical significance denoted as follows: $$^{*}$$$$p<0.05$$, $$^{**}$$$$p<0.01$$, $$^{***}$$$$p<0.001$$

Table 3 shows that in 2008 treated and control firms were similar along several firm dimensions, even though treated firms had higher profitability (higher ROA and Ebitda). During the pre-program implementation (2010–2013), both groups of firms were again comparable in several characteristics, but still, treated firms were more profitable, with higher sales and cash flows than control companies.

Thus, if one believes that part of the self-selection mechanism works through the unobserved ability of firms in proposing higher quality projects, and if this unobserved ability remained more or less constant through the sample period, then the Difference-in-Differences approach represents a credible estimation procedure in order to recover the causal of effect of interest. Indeed, the Difference-in-Differences methodology removes biases in the post-intervention period between the treatment and control groups that could be the result of permanent differences between those groups (such as ability, age, and gender), as well as biases from comparisons over time in the treatment group that could be the result of trends due to other causes of the outcome, despite the fact that this methodology is also a selection and self-selection robust approach.

The causal interpretation of the Difference-in-Differences estimate as the effect of the regional subsidy on the innovative investment level rests on the identifying assumption of (unconditional) parallel trends meaning that in the absence of the subsidy, eligible firms would have experienced the same average outcome variation in time (from pre-to-post-implementation) as firms with a score lower than 60 points. Therefore, my Difference-in-Differences approach estimates the causal effect of interest by comparing average expenditures in innovation activities of treated and control groups, identified by taking advantage of the assignment mechanism of the regional funds.

In particular, I estimated the following reduced form model that reads:

1 $$\begin{aligned} INVESTMENT_{i,t} = \beta SUBSIDY*POST + \lambda _{t} + f_{i} + \gamma X_{i,t} + \varepsilon _{i,t} \end{aligned}$$

where $INVESTMENT_{i,t}$ is the outcome of interest for firm *i* at time *t*, that is the yearly changes of tangible and intangible capital; *SUBSIDY* is a dummy that takes value of 1 if firm *i* is included in the treatment group, that is it received the subsidy or in other words it received a score of at least 60 points; *POST* is a dummy that takes values of 1 during the period of implementation of the program (2014–2015), and 0 otherwise. The coefficient of interest measuring the causal effect of the subsidy on the level of innovative expenditures is given by $\beta$. $\lambda _{t}$ are year fixed effects, $f_{i}$ are firms fixed effects. To improve estimates precision, the vector of controls includes cash flows and sales (since these are positively correlated with reported scores, see Table 4). Standard errors are clustered at the firm level (Bertrand et al. 2004).

**Table 4:** Correlation between Score (2013) and Observables (2008)

	(1)	(2)	(3)	(4)	(5)	(6)	(7)	(8)
INVESTMENT	− 0.00							− 0.00
	(0.00)							(0.00)
SALES		0.00$^{+}$						− 0.00
		(0.00)						(0.00)
CASH FLOWS			0.00$^{*}$					0.00
			(0.00)					(0.00)
LIQUIDITY				0.00				0.00
				(0.00)				(0.00)
SMALL					1.47$^{*}$			1.20
					(0.61)			(0.75)
MED-LARGE					2.47$$^{**}$$			1.93$$^{+}$$
					(0.86)			(1.14)
LOW-TECH						1.19$^{*}$		1.38$^{*}$
						(0.56)		(0.68)
MANUF.							− 0.53	− 1.68$^{*}$
							(0.63)	(0.77)
CONSTANT	59.72$$^{***}$$	59.33$$^{***}$$	59.40$$^{***}$$	59.43$$^{***}$$	58.71$$^{***}$$	58.83$$^{***}$$	59.64$$^{***}$$	58.77$$^{***}$$
	(0.32)	(0.33)	(0.30)	(0.32)	(0.37)	(0.43)	(0.33)	(0.50)
Observations	305	337	337	337	369	369	369	305
$R^{2}$	0.00	0.01	0.01	0.01	0.03	0.01	0.00	0.05

The dependent variable is given by the Score obtained after the projects’ technical evaluation in 2013. Score $$>60$$ implies that the firm is entitled to obtain the subsidy. Robust standard errors in parenthesis. Statistical significance denoted as follows: $$^{+}$$$$p<0.10$$, $$^{*}$$$$p<0.05$$, $$^{**}$$$$p<0.01$$, $$^{***}$$$$p<0.001$$

Having stated the reduced-form model, I would like to briefly discuss how to interpret the coefficient of the causal effect of interest. A coefficient $\beta >0$ can be interpreted as a signal of crowding-in, that is, treated firms invested additionally with respect to the control group because of the subsidy. On the contrary, a coefficient $\beta \le 0$ signals crowding-out, that is, treated firms substitute private funds with public capital. However, a simple $\beta >0$ statistically different from zero does not guarantee that the input-additionality holds, although the positive response of the investment level. To check if the hypothesis holds, I perform a simple hypothesis testing where the null ($H_{0}$) is given by ${\hat{\beta }}^{DD}\le$ threshold (that is, there is no evidence of input-additionality), whereas the alternative ($H_{1}$) is ${\hat{\beta }}^{DD}>$ threshold (input-additionality hypothesis holds). The threshold is given by the average value of the subsidy (obtained by treated firms, only) over the period 2014-2015, assuming that firms spent half of the public incentive in each year, that is half in 2014 and a half in 2015 (to be also consistent with the investment program).

**Table 5:** Baseline estimates

	(1)	(2)
SUBSIDY*POST	51,260.51$^{+}$	51,964.21$^{+}$
	(28,707.87)	(27,783.75)
CONTROLS	NO	YES
Observations	2,129	2,125
Adjusted $R^{2}$	0.13	0.15

Controls include cash-flows and revenues. Clustered robust standard errors at the firm level in parenthesis. Statistical significance denoted as follows: $$^{+}$$ $$p<0.10$$, $$^{*}$$$$p<0.05$$, $$^{**}$$$$p<0.01$$, $$^{***}$$$$p<0.001$$

## Main Results

*Baseline Estimates* Table 5 reports the estimated coefficients measuring the causal effect of interest, SUBSIDY*POST. Column (1) refers to the basic specification omitting control variables, whereas, Column (2) includes them. The sample period goes from 2010 to 2015, where the pre-treatment period is between 2010 and 2013. As previously highlighted, standard errors are clustered at the firm level to avoid potential serial correlation across periods (Bertrand et al. 2004).

According to the results, I find that the causal effect of the subsidy on the average level of expenditures in innovation is positive and statistically different from zero. The ATT amounts to 51,260.5 in Column (1) and to 51,964.2 in Column (2) with the inclusion of controls. These estimates suggest that subsidy recipient firms increased their innovative investment 1.5 times more than their level of spending in 2013.

In addition, to get a sense of the economic magnitude of these findings, I used a back-of-the-envelope calculation to estimate the elasticity of the investment to its price.^6^ The implied elasticity amounts to $-3$, a value that turns out economically meaningful despite being slightly above but sufficiently close to those estimated within the economic literature (see among the many Agrawal et al. 2020; Acconcia and Cantabene 2018).

Finally, to assess the fulfilment of the input-additionality hypothesis, I implement the test described in Sect. 3. In this case, the threshold value, given by the average value of the subsidy and assuming that public funds are spent equally between the two post-treatment years, is about 39,989.9 Euro. The t-test’s p-value is lower than 0.01, implying that that firms’ expenditures increased by more than the amount of the subsidy received, providing support for the input-additionality mechanism.

*Parallel Trends Assumption* The critical identification assumption of the Difference-in-Differences methodology is that, nevertheless differences in level, trends in outcome would be the same in both groups in the absence of the treatment, that is, the well-known common trend assumption or parallel trends assumption. Figure 2 shows that the average outcome has been almost identical for treated and control firms during the pre-implementation period. After the program begins, average expenditures have increased, but that of subsidy recipient firms more. However, despite this positive result based on descriptive evidence, a claim stating that parallel trends are accomplished would be misleading. Hence, to dissolve any doubts about its fulfilment, I estimate the baseline model by interacting the eligibility status indicator with year-dummies (from 2010 to 2015) while omitting 2013 as the reference category. That is, I consider the following equation, which consists of an event-study, that estimates the baseline regression with different treatment years:

2 $$\begin{aligned} INVESTMENT_{i,t} = \sum _{\tau =2010}^{2015} \gamma _{\tau } SUBSIDY*\mathbf{1} (t = \tau ) + \lambda _{t} + f_{i} + \omega X_{i,t} + \epsilon _{i,t}\nonumber \\ \end{aligned}$$

**Fig. 3:**
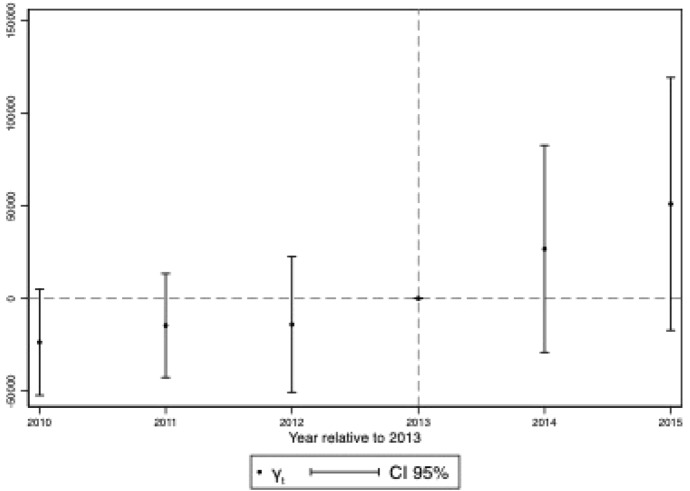
Event Study Estimates (Year Relative to 2013)

Equation 2 includes interactions between the treatment indicator variable (*SUBSIDY*) and year dummies for every year excluded 2013. Under the assumption of parallel trends $\gamma _{\tau }=0$ for $\tau < 2013$. Figure 3 reports the point estimates for $\gamma _{\tau }$ in Eq. 2 and $95\%$ confidence intervals. Given the long time span occurred between the issuing of the program and its final conclusion with the publication of the list of subsidy *recipients*, it may have been the case that some of the planned investments were conducted well before the timing set by the Region (01/01/2014–31/12/2015) and so if it is the case the *common trend assumption* is not satisfied. However, the Figure provides evidence of the absence of anticipation effects of the subsidy program. Furthermore, besides the lack of anticipation effects before year 2013 (year in which firms knew their final status) there is no evidence of statistically significant differences in outcome between control and treated groups, in other words there is no evidence of pre-trends, as the point estimates in the pre-period are not statistically different from zero. The point estimates of $\gamma _{\tau }$ for $\tau > 2013$ show the dynamics of the effect of the program. Unfortunately, despite an increasing profile of firms’ innovative investments, the estimates are never statistically different from zero. In addition, I test also the robustness of this result by varying the reference year of the event-study. Figure 7 reports the estimated coefficients when I consider as reference year 2010, 2011 and 2012. Overall, the pre-program coefficients are never statistically significant, even though their magnitude is mildly affected by the reference category considered. The post-program coefficients, again, are all positive and reporting similar magnitude to those in Fig. 3. Unfortunately, most of them are not statistically significant at the conventional confidence levels.

## Heterogeneity

So far, my estimates suggest a positive effect of the regional public contribution to the level of spending, being also compatible with the input-additionality hypothesis. However, the main findings may mask heterogeneity in the firms’ investment responses.

As a first heterogeneity exercise, I would like to understand whether different subsidy levels imply higher or lower innovative expenditures. In the flavour of the Dose-Response Model proposed by Cerulli (2015),^7^ I estimate Eq. (1) by replacing the treatment indicator (within the interaction term) with dummies taking value of 1 if the subsidy awarded falls within the first, the second, the third or the last quartile of its distribution.

**Table 6:** Heterogeneity by subsidy quartiles

	(1)	(2)
QUART. 1*POST	− 14,841.39	− 12,291.99
	(20,951.06)	(19,870.74)
QUART. 2*POST	28,378.57	27,213.59
	(37,968.05)	(36,752.31)
QUART. 3*POST	127,933.15$^{+}$	130,482.94$^{+}$
	(71,723.61)	(69,626.51)
QUART. 4*POST	63,167.23	61,923.17
	(51,931.37)	(54,931.12)
CONTROLS	NO	YES
Observations	2129	2,125
Adjusted $R^{2}$	0.14	0.15

Controls include cash-flows and revenues. Clustered robust standard errors at the firm level in parenthesis. Statistical significance denoted as follows: $$^{+}$$ $$p<0.10$$, $$^{*}$$$$p<0.05$$, $$^{**}$$$$p<0.01$$, $$^{***}$$$$p<0.001$$

The results of this test are available in Table 6. As usual, Column (1) reports the estimates without including controls, while Column (2) adds them. The estimates show that different subsidy levels contribute to diverse expenditures patterns in line with the initial guess. This relationship, however, is not monotonic but instead has an inverted U-shaped profile. I only find a statistically significant coefficient, although at the 10 percent level, for those firms whose public funds fall within the third quartile of the subsidy distribution. In relative terms, this coefficient translates into an average expenditures increase of $150\%$ and implied elasticity of 3 (identical to the baseline estimates). Furthermore, the estimated effect do support the input-additionality hypothesis since the t-test’s p-value is lower than 0.01.

The empirical micro-evidence on the effectiveness of public programs on innovation activities is not conclusive since the results are mixed and vary with the context (time period, country, industry; Klette et al. 2000), empirical approach (Cerulli 2010), outcome variables and level of government responsible for the policy program (David et al. 2000; Zúñiga-Vicente et al. 2014). The examination of the main findings corroborates the existence of a great diversity of results. Most of the empirical studies argue that the crowding-in mechanism crucially depends on the size of the firm, the volume of the public support (Görg and Strobl 2007) and the technological intensity of the sectors in which firms operate. The cases where public incentives tend to exhibit more input-additionality are relative to small firms (Lach 2002; González and Pazó 2008) operating in relatively low technological sectors (González and Pazó 2008; Becker and Hall 2013; see also Acconcia and Cantabene 2018) and firms located in less advantaged regions (Bronzini and de Blasio 2006). The majority of these works have mostly focused on the manufacturing sector. In general, manufacturing firms are characterized by a higher share of tangibles than intangibles, whereas the contrary is true for service firms. A *priori*, one can argue that programs that aim to sustain and increase innovation and technology intensity should be more effective for manufacturing firms because of their typical asset composition and reliance. However, the services sector has had an increasing and prominent weight in most developed countries. It is therefore important to analyze innovative investment in this sector, to understand how public subsidies affect it, and to compare the results with those for the manufacturing sector (Zúñiga-Vicente et al. 2014).

The size heterogeneity test is available in Table 7. I consider micro-sized firms to be those with fewer than ten employees on average between 2010 and 2013, small-sized firms to be those with 11–50 employees on average, and medium-large firms to be those with more than 50 employees on average. According to the results, only small-sized and medium-large firms significantly increase spending in innovation with an estimated coefficient of 104,695.2 and 199,411.4, respectively. The ATTs translate into an average increase in innovative investment of 100 percentage points with an implied elasticity of 2 for both sub-groups. Notably, I find also a negative but mildly statistically significant effect for micro-sized firms. However, I find that the spending increase fulfils the input-additionality hypothesis only for medium-large companies since the associated one-sided t-test’s p-value is statistically significant at the 1 percent level.

**Table 7:** Heterogeneity by firm size

	(1)	(2)
SUBSIDY*POST*MICRO	− 29,424.85$^{+}$	− 27,818.19$^{+}$
	(17,372.68)	(15,532.10)
SUBSIDY*POST*SMALL	101,496.09$^{+}$	104,695.23$^{*}$
	(53,947.01)	(52,322.37)
SUBSIDY*POST*MEDIUM-LARGE	210,010.22$^{*}$	199,411.38$^{*}$
	(89,180.70)	(98,426.40)
CONTROL	NO	YES
Observations	2,129	2,125
Adjusted $R^{2}$	0.15	0.16

Controls include cash-flows and revenues. Clustered robust standard errors at the firm level in parenthesis. Statistical significance denoted as follows: $$^{+}$$ $$p<0.10$$, $$^{*}$$$$p<0.05$$, $$^{**}$$$$p<0.01$$, $$^{***}$$$$p<0.001$$

I perform two sample-split exercises to further dig into the relationship between firm size and technology intensity. First, following the ATECO2007-NACE2 classification of the National Statistics Institute, I distinguish high and low-tech firms. Then, I divide the sample on whether firms operate in the manufacturing or service sectors. These tests are available in Tables 8 and 9. According to the evidence in Table 8, I find that the positive effect of the subsidy comes from low-tech medium-large enterprises (consistent with the crowding-in mechanism, p-value significant at the 5%). However, splitting the sample between manufacturing and service firms (Table 9) reveals that the whole effect is only marginally driven by low tech firms operating in the manufacturing sector. In addition, I also find a statistically significant coefficient regarding high tech medium large-sized firms operating in the service sector (a relative increase of 25 p.p. and an implied elasticity of 0.7, however, not compatible with the input-additionality hypothesis).

**Table 8:** Heterogeneity by firm size and sectors’ technology

	HIGH TECH.		LOW TECH.	
	(1)	(2)	(3)	(4)
SUBSIDY*POST*MICRO	− 10,363.20	− 7493.80	− 48,301.12	− 43,375.10
	(15,306.67)	(14,199.50)	(31,110.19)	(26,652.52)
SUBSIDY*POST*SMALL	81,821.58	51,309.26	103,696.48	109,391.60
	(68,366.22)	(45,160.38)	(77,016.70)	(75,011.46)
SUBSIDY*POST*MEDIUM-LARGE	− 21,005.19	− 57,504.68	227,999.43$^{*}$	226,543.14$^{*}$
	(64,487.26)	(88,066.98)	(102,593.97)	(107,012.73)
CONTROLS	NO	YES	NO	YES
Observations	972	968	1157	1157
Adjusted $R^{2}$	0.20	0.30	0.14	0.15

Controls include cash-flows and revenues. Clustered robust standard errors at the firm level in parenthesis. Statistical significance denoted as follows: $$^{+}$$ $$p<0.10$$, $$^{*}$$$$p<0.05$$, $$^{**}$$$$p<0.01$$, $$^{***}$$$$p<0.001$$

**Table 9:** Heterogeneity by firm size and sectors

	HIGH TECH.				LOW TECH.			
	MANUF.		SERVICE		MANUF.		SERVICE	
	(1)	(2)	(3)	(4)	(5)	(6)	(7)	(8)
SUBSIDY*POST*MICRO	1,504.95	23,204.33	− 12,008.02	− 13,079.91	− 40,593.86	− 21,757.92	− 49,253.45	− 50,998.43
	(69,764.25)	(59,098.08)	(11,488.40)	(11,663.37)	(57,811.34)	(37,972.87)	(33,796.18)	(34,225.00)
SUBSIDY*POST*SMALL	294,500.64	197,817.40	14,019.49	12,818.24	133,806.04	153,636.76	69,672.32	65,121.50
	(251,140.06)	(184,840.96)	(21,858.88)	(20,442.56)	(136,027.57)	(129,798.47)	(80,085.78)	(80,364.52)
SUBSIDY*POST*MEDIUM-LARGE	− 63,744.73	− 152,108.47	38,294.54$$^{***}$$	45,317.07$$^{***}$$	320,264.09	363,147.36$$^{+}$$	165,113.19$$^{+}$$	138,531.98
	(100,723.70)	(161,196.31)	(10,550.87)	(9860.10)	(210469.93)	(216,595.70)	(95,058.55)	(89,219.99)
CONTROLS	NO	YES	NO	YES	NO	YES	NO	YES
Observations	196	196	756	752	465	465	676	676
Adjusted $R^{2}$	0.20	0.39	0.23	0.24	0.14	0.15	0.09	0.09

Controls include cash-flows and revenues. Clustered robust standard errors at the firm level in parenthesis. Statistical significance denoted as follows: $$^{+}$$ $$p<0.10$$, $$^{*}$$$$p<0.05$$, $$^{**}$$$$p<0.01$$, $$^{***}$$$$p<0.001$$

## Robustness

As explained in Sect. 3, the natural empirical strategy to estimate the causal effect of interest would have been a Regression Discontinuity Design. However, as previously discussed, this method is unfeasible in my setting. Nonetheless, despite not relying on an RD specification, I can take advantage of the intuition behind the continuity assumption. The goal is to check whether, still within a Difference-in-Differences approach, the previous set of estimates remains stable, even shrinking the sample size around the cut-off (score = 60), alleviating any concern about the treatment status endogeneity. If this is the case, this means that the effect is not driven by firms with higher reported scores (and thus presumably higher quality), providing further validity to the empirical strategy adopted.

**Fig. 4:**
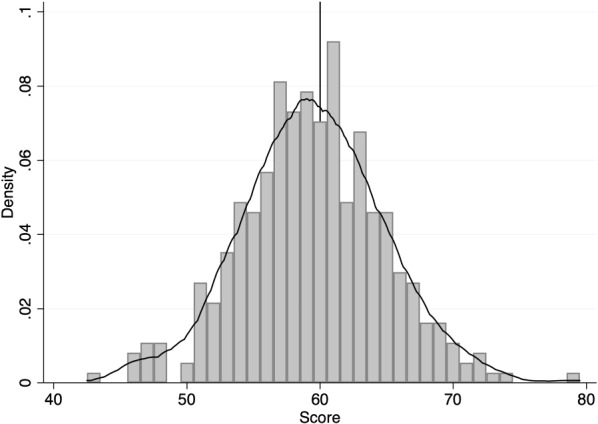
Firms’ Density Distribution by Score

**Fig. 6:**
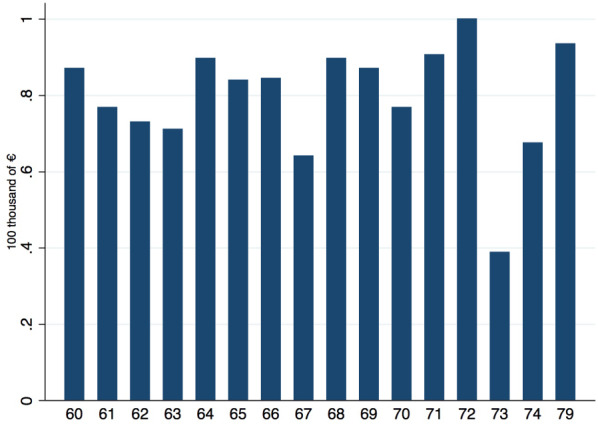
Average subsidy awarded by score. the lowest score in order to be eligible for obtaining the subsidy is 60 pts. The figure shows the average subsidy grant by score to eligible firms in thousands of Euro. The maximum amount of the subsidy that the Regional Government was allowed to award is set at 200,000 €, so that it does not break the State Aid Legislation

**Fig. 7:**
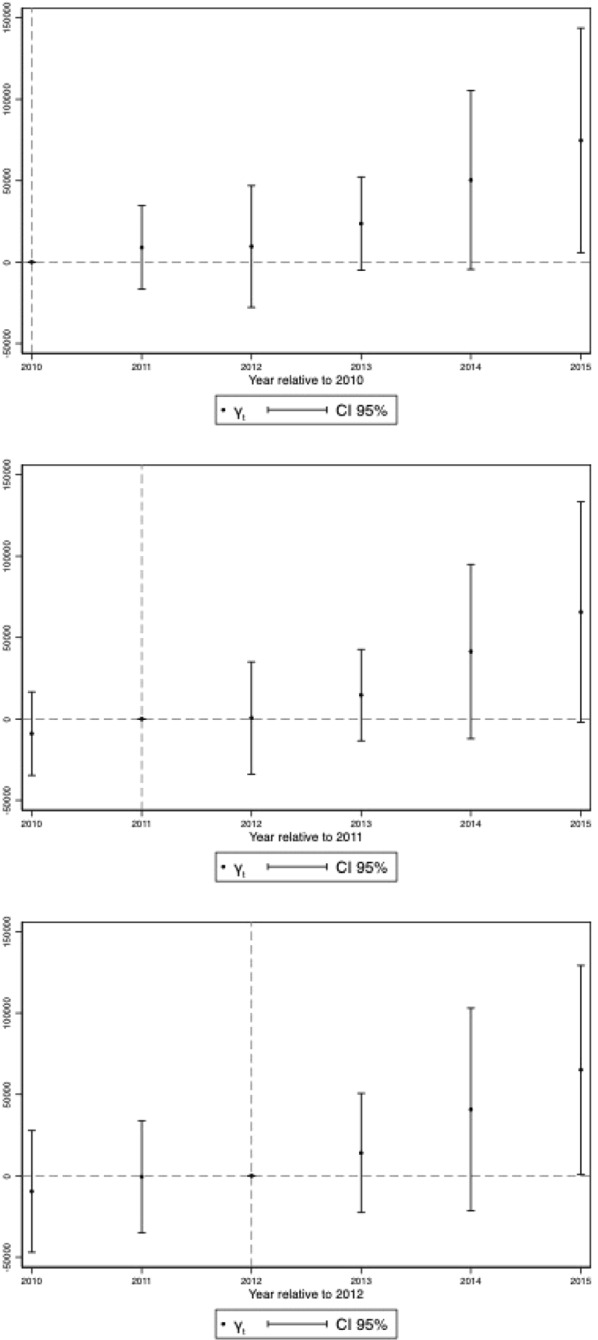
Event-study robustness

First, I discuss that indeed firms had no room to manipulate the forcing variable, as well there is no evidence that Technical Commission accommodated more firms in obtaining the public funds by assigning them the lowest score possible to get access to the subsidy. These conclusions come from the visual inspection of the density function of the sample by score (Fig. 4). Indeed, no evident excess of mass at the threshold, neither to the right nor to the left of it. This result is somewhat in favour of ruling out any possible sorting behaviour from both firms and the panel of experts that evaluated the investment projects.

To perform this robustness test, I estimate Eq. (1) and its modifications by selecting three different tighter windows around the eligibility threshold: [53–66], [56–64] and [58–62] windows.

According to the evidence in Tables 10 and 11, these robustness tests provide sufficient support to the relevant coefficients previously discussed in magnitude and statistical significance even in tighter bandwidth sizes around the cut-off in the majority of the cases. Diversely, for all those non statistically significant coefficients, the reported magnitude is different from the baseline estimates but still non statistically significant at the conventional confidence levels.

## Additional Effects

So far, my estimates show that the subsidy program has induced a sizeable increase in innovation expenditures, (partially) fulfilling the standard additionality criteria studied and advocated by the literature. Then, it is reasonable to inquire whether the program had indirect effects on treated firms.

**Table 10:** Robustness around the threshold

SCORE:	[53–66]		[56–64]		[58–62]	
	(1)	(2)	(3)	(4)	(5)	(6)
SUBSIDY*POST	44,172.09	44,661.02	− 4921.13	− 2864.95	49,389.85	59,352.56$^{+}$
	(33,462.75)	(32,512.25)	(34,573.25)	(32,259.42)	(29,842.35)	(35,132.55)
Observations	1754	1750	1308	1304	785	785
Adjusted $R^{2}$	0.14	0.16	0.13	0.14	0.14	0.15
SUBSIDY*POST*MICRO	− 34,215.57$^{+}$	− 32,390.29$^{+}$	− 52,618.59$^{+}$	− 50,829.11$^{*}$	5112.42	14,998.90
	(20,506.13)	(18,388.18)	(27,496.39)	(24,452.18)	(16,439.12)	(20,446.97)
SUBSIDY*POST*SMALL	101,813.37	104,701.30	2055.67	6,679.29	23,497.29	33,479.03
	(66,377.91)	(64,440.82)	(50,073.92)	(47,882.57)	(29,850.89)	(32,528.67)
SUBSIDY*POST*MEDIUM-LARGE	184,978.18$^{+}$	173,480.63	188,748.00	184,427.18	290,859.00$^{+}$	313,300.86$^{+}$
	(96,203.70)	(109,447.45)	(127,098.96)	(139,817.37)	(168,749.76)	(160,537.96)
Observations	1754	1750	1308	1304	785	785
Adjusted $R^{2}$	0.16	0.17	0.14	0.15	0.18	0.19
HIGH TECH.:						
SUBSIDY*POST*MICRO	− 6,657.03	− 1592.77	− 10,805.33	− 10,223.08	3315.75	3858.05
	(18,204.49)	(16,818.93)	(19,586.54)	(19,096.31)	(27,369.39)	(27,334.00)
SUBSIDY*POST*SMALL	137,511.59	85,983.40	67,050.87	63,927.36	39,079.39	36,174.56
	(92,468.48)	(60,635.44)	(51,086.30)	(51,669.62)	(40,555.93)	(39,485.70)
SUBSIDY*POST*MEDIUM-LARGE	− 21,598.75	− 34,494.57	− 71,831.06	15,704.18	− 68,712.51	− 39,755.88
	(65,171.88)	(77,751.45)	(76,139.30)	(68,428.11)	(78,561.92)	(87,477.57)
Observations	787	783	577	573	354	354
Adjusted $R^{2}$	0.24	0.35	0.20	0.22	0.30	0.30
LOW TECH.:						
SUBSIDY*POST*MICRO	− 58,801.02$^{+}$	− 53,360.94$^{+}$	− 88,357.76$^{+}$	− 82,834.66$^{*}$	− 2001.02	17,465.35
	(35,252.24)	(30,621.69)	(48,103.38)	(41,660.29)	(15,471.46)	(31,237.12)
SUBSIDY*POST*SMALL	73,013.85	81,441.48	− 45,233.72	− 34,360.00	25,880.52	39,386.59
	(90,385.42)	(87,978.31)	(74,497.71)	(69,106.75)	(36,504.68)	(42,274.07)
SUBSIDY*POST*MEDIUM-LARGE	204,047.62$^{+}$	204,062.49$^{+}$	201,733.89	215,977.74	390,891.25$^{+}$	399,070.06$^{*}$
	(113,631.90)	(120,648.64)	(147,756.66)	(154,415.48)	(200,079.78)	(188,872.02)
Observations	967	967	731	731	431	431
Adjusted $R^{2}$	0.14	0.15	0.13	0.14	0.17	0.17
CONTROLS	NO	YES	NO	YES	NO	YES

Controls include cash-flows and revenues. Clustered robust standard errors at the firm level in parenthesis. Statistical significance denoted as follows: $$^{+}$$ $$p<0.10$$, $$^{*}$$$$p<0.05$$, $$^{**}$$$$p<0.01$$, $$^{***}$$$$p<0.001$$

**Table 11:** Robustness around the threshold and sectors

SCORE:	[53–66]		[56–64]		[58-62]	
	(1)	(2)	(3)	(4)	(5)	(6)
*HIGH TECH. MANUF. SECTOR*						
SUBSIDY*POST*MICRO	− 27,476.73	35,705.18	36,363.33	68,344.75	110,015.39	113,464.23
	(117,942.60)	(100,371.55)	(77,921.76)	(61,225.65)	(87,931.30)	(93,227.01)
SUBSIDY*POST*SMALL	475,953.09	339,880.34	287,882.25	339,023.00		
	(342,615.27)	(268,081.89)	(228,103.16)	(259,845.15)	(.)	(.)
SUBSIDY*POST*MEDIUM-LARGE	− 92,350.91	− 88,419.94	− 147,035.75$$^{***}$$	257,446.52	− 147,156.25$$^{***}$$	− 199,116.29
	(139,117.94)	(176,519.60)	(8694.99)	(586,605.71)	(11,342.99)	(180,211.76)
Observations	125	125	83	83	53	53
Adjusted $R^{2}$	0.28	0.42	0.03	0.07	0.17	0.12
*HIGH TECH. SERVICE SECTOR*						
SUBSIDY*POST*MICRO	− 6785.55	− 8118.35	− 17,427.71	− 18,739.99	− 17,154.58	− 23,388.86
	(12,384.37)	(12,657.81)	(17,172.27)	(17,276.63)	(26,862.11)	(27,878.66)
SUBSIDY*POST*SMALL	32,626.90	26,689.39	25,068.08	18,015.11	34,391.27	17,477.21
	(25,904.86)	(24,701.91)	(31,930.34)	(30,765.19)	(43,049.41)	(43,581.54)
SUBSIDY*POST*MEDIUM-LARGE	38,883.34$$^{**}$$	50,165.97$$^{***}$$	28,806.16$$^{+}$$	39,900.67$$^{**}$$	30,865.37	46,213.80$$^{*}$$
	(11,779.38)	(10,257.86)	(16,733.46)	(14,369.21)	(26,687.80)	(20,805.22)
Observations	642	638	488	484	301	301
Adjusted $R^{2}$	0.29	0.30	0.30	0.30	0.33	0.34
*LOW TECH. MANUF. SECTOR*						
SUBSIDY*POST*MICRO	− 40,040.14	− 17,033.86	− 74,849.56	− 33,190.36	8,102.21	19,418.79
	(68,611.89)	(48,065.36)	(108,745.83)	(73,288.93)	(15,021.60)	(35,180.32)
SUBSIDY*POST*SMALL	75,012.10	97,324.28	− 16,0103.27	− 121,513.78	10,897.31	40,118.24
	(147,803.98)	(140,700.99)	(118,962.26)	(89,475.93)	(75,354.58)	(89,029.15)
SUBSIDY*POST*MEDIUM-LARGE	355,297.10	406,746.15$^{+}$	381,463.73	433,557.80	543,996.66$^{+}$	550,785.16$^{+}$
	(233,422.66)	(242,950.25)	(272,560.63)	(282,495.71)	(313,706.52)	(283,879.36)
Observations	387	387	251	251	138	138
Adjusted $R^{2}$	0.14	0.16	0.15	0.17	0.16	0.16
*LOW TECH. SERVICE SECTOR*						
SUBSIDY*POST*MICRO	− 56,144.36	− 58,087.29	− 81,408.98$^{+}$	− 85,461.31$^{+}$	− 4254.72	− 13,812.34
	(38,495.91)	(38,925.84)	(47,527.33)	(47,760.68)	(18,786.24)	(18,917.65)
SUBSIDY*POST*SMALL	62,338.55	60,380.02	46,718.30	41,105.45	37,324.25$^{+}$	29,488.83
	(104,229.62)	(104,384.39)	(114,431.60)	(114,374.15)	(20,432.89)	(19,546.07)
SUBSIDY*POST*MEDIUM-LARGE	83,454.10	52,740.53	3,916.28	− 3,591.57	155,185.48$^{+}$	145,491.50$^{+}$
	(67,596.10)	(55,219.55)	(70,120.48)	(69,177.61)	(87,510.48)	(86,116.16)
Observations	564	564	464	464	283	283
Adjusted $R^{2}$	0.08	0.09	0.08	0.08	0.08	0.09
CONTROLS	NO	YES	NO	YES	NO	YES

Controls include cash-flows and revenues, both scaled by Total assets. Clustered robust standard errors at the firm level in parenthesis. Statistical significance denoted as follows: $$^{+}$$$$p<0.10$$, $$^{*}$$$$p<0.05$$, $$^{**}$$$$p<0.01$$, $$^{***}$$$$p<0.001$$

The first additional effect I investigate is firms’ labour demand. According to official figures retrieved from the subsidy recipients project forms, 55% of treated firms in 2009 would have been willing to hire new employees following the innovative investment. For clarity, however, the subsidy program did not cover wage expenditures nor aimed at inducing firms to increase employment to get access to public funds.

Hence, by exploiting Eq. (1) within its heterogeneous specification, I test whether public funds awarded to firms increased employment in the aftermath of the program relative to control firms. Unfortunately, I do not have access to matched employer-employee administrative data, and I am forced to analyse the aggregate workforce reported at the end of the year in financial statements.

However, one of the limitations of this employment measure is that it is expressed in terms of full-time equivalent units. To overcome this issue, I use as an additional dependent variable the per worker wage bill that may be more sensitive to changes in the overall worker composition.^8^

As a second potential indirect effect, I study the impact of the subsidy on two productivity measures common in the accounting literature, which are added value per worker and added value over Total asset.

**Table 12:** Additional effects and heterogeneity

	HIGH TECH.							
	MANUF.				SERVICE			
	NO. WORKER	STAFF COSTS	AVPW	AV/TA	NO. WORKER	STAFF COSTS	AVPW	AV/TA
SUBSIDY*POST*MICRO	− 0.64	746.00	152.01	0.85	26.09	− 17381.80	− 19,743.58	0.02
	(2.67)	(2,485.30)	(7891.31)	(0.88)	(24.93)	(12,591.70)	(19,883.85)	(0.04)
SUBSIDY*POST*SMALL	− 9.60$^{*}$	11,219.43	20,097.56	0.78	40.10	− 42,935.29$^{+}$	− 66,219.71$^{+}$	− 0.05
	(3.52)	(6747.25)	(20,630.93)	(0.88)	(28.30)	(22,309.09)	(36,121.47)	(0.12)
SUBSIDY*POST*MEDIUM-LARGE	18.21	7604.50	10,191.42	0.84	24.19	− 15,390.25	− 24,802.35	0.02
	(17.33)	(4850.02)	(7192.78)	(0.88)	(25.61)	(12,879.67)	(18,716.00)	(0.02)
CONTROLS	NO	YES	NO	YES	NO	YES	NO	YES
Observations	187	166	166	198	734	579	584	772
Adjusted $R^{2}$	0.83	0.51	0.69	0.05	0.40	0.33	0.29	0.73

	LOW TECH.							
	MANUF.				SERVICE			
	NO. WORKER	STAFF COSTS	AVPW	AV/TA	NO. WORKER	STAFF COSTS	AVPW	AV/TA
SUBSIDY*POST*MICRO	1.11	− 8383.31$^{+}$	4850.50	0.10$^{+}$	− 3.29$^{+}$	2,864.81	13,024.61	0.02
	(4.25)	(4490.01)	(19,406.95)	(0.06)	(1.76)	(4,101.52)	(14,752.74)	(0.03)
SUBSIDY*POST*SMALL	− 0.02	− 9386.24$^{*}$	4,647.00	0.05	− 1.22	4,205.48	12,192.16	0.04
	(4.46)	(4,406.54)	(20,671.67)	(0.03)	(2.54)	(4,092.11)	(14,358.85)	(0.03)
SUBSIDY*POST*MEDIUM-LARGE	5.55	− 10,606.18$^{*}$	− 21,747.57	0.04	50.25$^{*}$	3,353.78	5,134.49	0.08$^{+}$
	(14.07)	(4,370.99)	(26,053.19)	(0.04)	(21.36)	(3,954.87)	(14,494.81)	(0.05)
CONTROLS	NO	YES	NO	YES	NO	YES	NO	YES
Observations	458	436	436	473	666	593	593	684
Adjusted $R^{2}$	0.77	0.26	0.44	0.80	0.84	0.19	0.32	0.79

Controls include cash-flows and revenues, both scaled by Total assets. Clustered robust standard errors at the firm level in parenthesis. Statistical significance denoted as follows: $$^{+}$$$$p<0.10$$, $$^{*}$$$$p<0.05$$, $$^{**}$$$$p<0.01$$, $$^{***}$$$$p<0.001$$

Table 12 reports the results for labour demand, per-capita wage bill, per-capita added value and added value or total assets. The first panel refers to high tech firms, while the latter one to low tech companies. However, both distinguish the sample according to firms operating in the manufacturing or the service sector.

Concerning labour demand, I find that high tech manufacturing small-sized firms and low tech service micro-sized companies (although the point estimate is only statistically significant at 10 percent) reduce labour demand. On the contrary, low tech service medium-large companies increase significantly employment, a relative effect of $+45\%$. Furthermore, all low tech manufacturing firms decrease their per-capita wage costs, potentially suggesting labour demand substitution towards cheap labour by keeping their overall number of employees almost constant.

Regarding firms’ productivity, I do not find any particular beneficial effect of the subsidy. In the majority of the cases, estimates are not statistically significant, alternating in sing and not being very clear on true effect.

## Conclusions

This paper provides novel empirical evidence on the effectiveness of public funding for innovative private investment in lagging behind regions. In particular, my analysis leverages a subsidy investment program implemented in the Campania region during 2014–2015, targeting small and medium enterprises (SMEs).

My estimates show a sizable innovative investment increase of subsidy-recipient firms relative to non-eligible companies after the program implementation. In addition, I also document large heterogeneity in the firms’ responses. The analysis reveals that different subsidy intensities contribute to an inverted U-shaped response in eligible firms capital investment. Furthermore, diversely from previous evidence, I find that only medium-large firms and medium-large low-tech firms increased spending above the size of the subsidy received (thus, compatible with the input-additionality hypothesis). Finally, I show considerable indirect effects on medium-large firms labour demand operating in the service sector but not overall improvements in firms’ productivity.

On the policy side, these findings reinforce the argument for which these programs are more effective if directed to enterprises operating in traditional sectors whose innovation projects are more likely to be subject to liquidity constraints and asymmetric information on financial markets. This evidence, further, seems to support the view that a quick and robust administrative capacity should support public funding programs aimed at increasing spending in innovation activities to ensure that such policies can be conceived as effective instruments in stimulating investment demand, especially during downturn business cycle periods.

## Figures and Tables

**Fig. 5:**
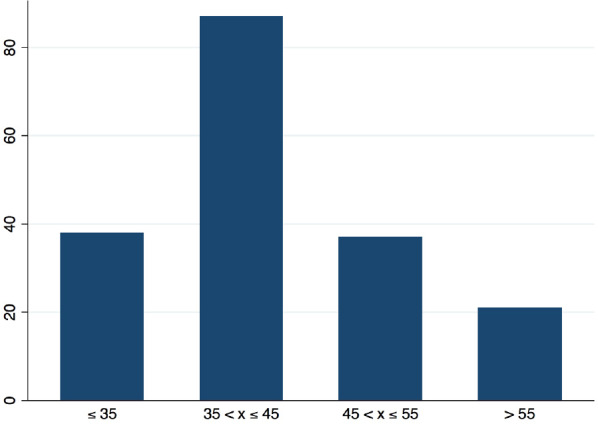
Number of subsidized project by age groups

## A. The Regional Program: Additional Details

The maximum scores attributed to each macro-criteria summarized in Table 1 are defined as follows:

1. **Quality and innovation of the project, both for the purpose of increasing the efficiency of the management machine, and as a function of completing/upgrading existing ICT infrastructures** (*max* 60 pts):
  - Quality of the project in terms of precision and completeness in the identification of specific actions to be carried out, with particular regard to organizational and management procedures: *max* 20/60 pts
  - Innovation on the production organization: incidence of the interventions to be carried out on the strengthening of the production chain activity (transformation plants, company sales points, introduction and/or e-commerce development): *max* 20/60 pts
  - If the project is to complete/upgrade existing **ICT** infrastructures: *max* 10/60 pts
  - If the project involves the improvement of the company organization (reduction of the company underemployment, reconversion and / or increase in employment,$\ldots$) and of safety in the workplace: *max* 10/60 pts
2. **Impact on the qualification of the product/service with a relative increase of competitiveness on the market** (*max* 30 pts):
  - If the project involves the creation of new products and/or the diversification of some others and/or the quality certification of the company productions/services: *max* 10/30 pts
  - Percentage increase in the expected corporate added value with the measures co-financed when fully operational: *max* 10/30 pts
  - Economic sustainability, deductible from the relationship between total cost of the project and annual company sales volume: *max* 5/30 pts
  - Environmental sustainability, in the presence of interventions and/or machinery that reduce pollutant emissions or improve the management of company waste: *max* 5/30 pts
3. **Relevance of the juvenile and female components** (*max* 10 pts):
  - Age of the applicant (individual company), average age of members (partnership) of the Sole Administrator or average of members of the board of directors (limited liability company): $\le 35$ years 7/10 pts; $35<x\le 45$ years 5/10 pts; $45<x\le 55$ years 3/10 pts e $>55$ years 1/10 pts
  - Applicant sex (individual company), prevalent sex of members (partnership), of the sole director or predominantly of the members of the board of directors (joint-stock company): if female 3/10 pts

For the purpose of compiling the final merit ranking, the total score assigned to each project will be determined by the sum of the scores assigned for each of the evaluation parameters for a maximum of 100 pts. If the resulting sum will be less than 60 pts, the project will not be included in the final ranking.

**Table 13:** Summary statistics by age and sex of treated firms’ stakeholders

	OBS	Mean	SD	Min	Max
Age	183	42.3	9.72	19	75
Prevalent sex	183	.76	.43	0	1

Data about age and prevalent sex of firms’ stakeholders are only available for treated firms. The variable Age is expressed in years, whereas the variable prevalent sex is a dummy variable that takes value of 1 if the average sex of the relevant stakeholder in *recipient* firm is male

## B. Additional Tables and Figures

**Table 14:** Data retrieved from AIDA DB

	Not admitted			Admitted								
	to evaluation			to evaluation								
	(N=1354)			(N=820)								
				Non recipients			Recipients:			Renunciations:		
				score $<60$			score $\ge 60$			score $\ge 60$		
				(N = 424)			(N = 299)			(N=97)		
	(A)	(B)	(C)	(A)	(B)	(C)	(A)	(B)	(C)	(A)	(B)	(C)
	Recorded in	Bankruptcy	No longer	Recorded in	Bankruptcy,	No longer	Recorded in	Bankruptcy	No longer	Recorded in	Bankruptcy	No longer
	AIDA	liquidation,	recorded in	AIDA	liquidation,	recorded in	AIDA	liquidation,	recorded in	AIDA	liquidation,	recorded in
		dissolution	AIDA		dissolution	AIDA		dissolution	AIDA		dissolution	AIDA
31/12/2009	838	13	11	313	1	2	232	0	0	74	0	0
31/12/2013	683	108	47	251	45	17	220	4	7	46	23	5
31/12/2016	521	84	78	188	35	28	185	19	17	24	13	9

At 31/12/2009, 2174 applications were received for participation in the call for tenders announced by the *Campania* Region for the allocation of capital grants (subsidies) for the implementation of innovative investments by local SMEs. Of these, 1354 applications were excluded from the subsequent phase of project evaluation, as these companies were non-compliant with program’s regulation due to the lack of presentation of some preliminary documents for the participation of the tender. 820, instead, were the proposals admitted to the evaluation phase, which took place between 2011 and 2013. The values included in the table refer to the availability in the AIDA database of the last deposited balance sheet: at the conclusion of the submissions of the applications for participation, at the time of publication of the final ranking of participants (*Eligible assignees and Eligible non-assignees*), 1 year from the deadline for the realization of investments. In particular, starting from the year 31/12/2013 and summing the columns (A, Recorded in AIDA), (B, Bankruptcy, Liquidation, Dissolution) and (C, No longer registered in AIDA) we obtain the number of enterprises *Recorded in AIDA* (column “A”) referring to the previous period indicated in the table. For example, for Not admitted to evaluation as of 31/12/2009 these are 838; adding the line referring to 31/12/2013, i.e. $$ 683+108+47$$ we obtain the number of companies registered in AIDA at 31/12/2009 for the relative *sub-group*. The number of reported BS is reduced over time due to the modification of the legal status of the participating companies (declaration of bankruptcy, dissolution, entry into liquidation) or, although still active, no longer recorded by AIDA.

**Table 15:** Balancing at threshold: $t=$ 2010–2013

	CONTROL	OBS.	TREATED	OBS.	$\Delta$
*SCORE: 53–66*					
INVESTMENT	34,211.99	584	32,636.39	580	1,575.60
LEVERAGE	7.95	595	6.82	584	1.13
COST OF DEBT	7.75	327	7.52	338	0.23
ROA	4.00	598	5.63	584	− 1.63$$^{**}$$
SALES	4,163,506.79	598	6,032,572.49	584	− 1,869,065.70$$^{**}$$
EBITDA	284,142.66	595	581,398.53	584	− 297,255.88$$^{***}$$
CASH FLOWS	163,700.87	595	378,522.97	584	− 214,822.10$$^{***}$$
LIQUIDITY	268,233.42	595	353,148.57	584	− 84,915.15
NO. WORKERS	30.26	561	23.01	554	7.25

*SCORE: 56–64*					
INVESTMENT	29,979.03	398	36,032.38	468	− 6053.35
LEVERAGE	7.45	406	7.12	472	0.33
COST OD DEBT	7.65	229	7.67	266	− 0.03
ROA	4.25	409	5.60	472	− 1.35$^{*}$
SALES	4,382,170.14	409	5,799,930.42	472	− 1,417,760.28$^{*}$
EBITDA	301,702.86	406	590,724.98	472	− 289,022.11$$^{**}$$
CASH FLOWS	173,616.50	406	381,703.04	472	− 208,086.54$$^{**}$$
LIQUIDITY	293,178.70	406	331,589.67	472	− 38,410.97
NO. WORKERS	36.86	384	19.51	449	17.35

*SCORE: 58–62*					
INVESTMENT	25,980.23	212	37,943.49	308	− 11,963.26
LEVERAGE	4.73	216	7.44	310	− 2.71
COST OD DEBT	8.42	119	7.53	168	0.89
ROA	3.62	216	5.02	310	− 1.40$$^{**}$$
SALES	3,432,669.04	216	5,472,773.73	310	− 2,040,104.69$^{*}$
EBITDA	186,323.51	216	450,495.83	310	− 264,172.32$$^{**}$$
CASH FLOWS	91,359.66	216	289,738.94	310	− 198,379.27$^{*}$
LIQUIDITY	155,062.20	216	266,778.57	310	− 111,716.36
NO. WORKERS	18.87	199	19.83	294	− 0.96

Statistical significance denoted as follows: $$^{*}$$$$p<0.05$$, $$^{**}$$$$p<0.01$$, $$^{***}$$$$p<0.001$$

**Table 16:** Firms composition by size per year

	FREQ.	$\%$	CUM. $\%$	TREATED	CONTROL
MICRO	203	55.01	55.01	90	113
SMALL	128	34.60	89.61	68	60
MEDIUM-LARGE	38	10.39	100	24	14
Total	367	100		181	186

Firms size decomposition follows the definition given by the EU Commission. In particular, I consider micro-sized firms those up to 10 workers on average during the pre-*treatment* period (2010–2013), small-sized those between 11 and 50 workers on average, medium-sized those between 51 and 250 and large-sized those with more than 250 workers on average

**Table 17:** High and low tech NACE-ATECO2007 2-digits sectors

2-digits code	Description	Type
*HIGH TECH*		
26	Electronics and components	M
27	Electric devices	M
28	Machines and mechanical devices	M
29	Automotive	M
30	Means of transport	M
32	Medical devices	M
33	Maintenance of machines	M
35	Energy	M
58, 62, 63	Information and communication services	S
64, 65	Financial services	S
66	SME auxiliary financial services	S
68-71	Consultancy	S
72	Research and development	S
73, 74	Market research and other professional activities	S
77, 78, 80–82	Firms’ services	S
85	Education	S

*LOW TECH*		
10, 11	Foods, beverage and tobacco	M
14, 15	Clothing and apparel	M
17	Paper products	M
18	Printed products	M
22	Articles in gum and plastic	M
23	Glass and concrete products	M
24	Iron and steel industry	M
25	Metallic constructions	M
38	Water and environment industry	M
41–43	Construction sector	M
45	Automotive commercial services	S
46	Wholesale services	S
47	Retailing services	S
49	Terrestrial transport services	S
52	Logistic services	S
55	Catering services	S
56	Hotel and tourism industry	S
79	Tourism services	S
86	Healthcare services	S
88	Non-residential social assistance	S
90	Entertainment services	S
93	Sport activities and services	S

Type: M stands for Manufacturing sector; S for Service sector. Source: Firms technology intensity by sector based on ATECO 2007 2-digits classification according to technology intensity across economic sectors available at: https://www.istat.it/it/files//2017/08/GlossarioNotaMetodologica.pdf

## Footnotes

1 For a detailed overview of the criteria set out by the Region for the eligibility status consult Sect. 1 of the Appendix.

2 The exclusion of firms from having access to the public support measure regarded the non-compliance of them with all the documentation requested by the region to be attached to the submission files.

3 The BURC is the information press service that advertises the laws, regulations and acts of the region, assuming a role similar to that performed by the Official Gazette (Gazzetta Ufficiale). In the legal field, it is considered as one of the official sources of legal knowledge.

4 An ERP system is the integrated management of core business processes, often in real-time and mediated by software and technology that provides the infrastructure to manage information and coordinate activities within the firm to develop more efficient operations and to take advantage of benefits in terms of cost and time saving, routines and information exchange within the firm.

5 In constructing my dependent variable, I had to overcome another issue. The Italian regulation allows certain types of firms to file a simplified balance sheet scheme, where most of the items are grouped in broad categories. Up to $44\%$ of the sample files balance sheet under the classic scheme, while the remaining $56\%$ reports a generic voice of Intangibles. However, I can subtract the accounting value of goodwill and other intangibles from this broad item. To check the accuracy of this (net) measure in capturing only those intangibles that qualify for the regional funds, I computed it also for companies that file a classic financial statement. In $96\%$ of the cases, the match between the reconstructed measure from the broad category and actual values drawn from the balance sheet is perfect. Consequently, this confirms that even in the absence of detailed data, I can recover the intangibles book values of interest correctly. Finally, that is also why I cannot compute my investment measure as the variation in stocks plus flows (*i*.*e*., $R \& D$ expenditure amount) or as the (pre-post) cumulative investment avoiding stocks.

6 The implied elasticity is computed according to the following formula: $({\hat{\beta }}^{DD}/{\bar{INV}}_{2013}^{TREATED})*(1/SUBSIDY)$.

7 Unfortunately, I cannot rely on this specification given that regional funds received by firms have a cross-sectional nature, whereas the outcome variable has a panel structure because firms have to develop innovative investment during 2014–2015.

8 Indeed, it may be the case that firms may substitute expensive workforce with cheaper ones by relying on flexible job contracts and keeping constant the overall number of employees.
